# The Effect of Fat Distribution on the Inflammatory Response of Multiple Trauma Patients—A Retrospective Study

**DOI:** 10.3390/life11111243

**Published:** 2021-11-16

**Authors:** Zhaoxiong Chen, Silvan Wittenberg, Timo Alexander Auer, Maxim Bashkuev, Pimrapat Gebert, Uli Fehrenbach, Dominik Geisel, Frank Graef, Sven Maerdian, Serafeim Tsitsilonis

**Affiliations:** 1 Center for Musculoskeletal Surgery, Charité - Universitätsmedizin Berlin, Corporate Member of Freie Universität Berlin and Humboldt-Universität zu Berlin, Berlin Institute of Health, 13353 Berlin, Germany; zhaoxiong.chen@charite.de (Z.C.); silvan.wittenberg@charite.de (S.W.); frank.graef@charite.de (F.G.); 2 Clinic for Radiology, Charité - Universitätsmedizin Berlin, Corporate Member of Freie Universität Berlin and Humboldt-Universität zu Berlin, Berlin Institute of Health, 13353 Berlin, Germany; timo-alexander.auer@charite.de; 3 Julius Wolff Institute for Biomechanics and Musculoskeletal Regeneration, Charité - Universitätsmedizin Berlin, 10115 Berlin, Germany; maxim.bashkuev@charite.de; 4 Institute of Biometry and Clinical Epidemiology, Charité - Universitätsmedizin Berlin, 10115 Berlin, Germany; pimrapat.gebert@charite.de; 5 Clinic for Radiology, Charité - Universitätsmedizin Berlin, Corporate Member of Freie Universität Berlin and Humboldt-Universität zu Berlin, 13353 Berlin, Germany; uli.fehrenbach@charite.de (U.F.); dominik.geisel@charite.de (D.G.)

**Keywords:** fat distribution, systemic inflammatory response syndrome, polytrauma, image segmentation

## Abstract

Objectives In recent years; increasing evidence pointed out the clinical importance of adipose tissue (AT) distribution in various patient populations. In particular, visceral adipose tissue (VAT), when compared to subcutaneous adipose tissue (SAT), was found to play a pivotal role in the development of inflammatory reaction. The aim of the present study was to examine whether body fat distribution has an impact on the development of systemic inflammatory response syndrome (SIRS) in patients with polytrauma. Methods In our retrospective study; we filtered our institution records of the German Trauma Registry (Trauma Register DGU) from November 2018 to April 2021 and included 132 adult polytrauma patients with injury severity score (ISS) >16. Subsequently; we measured the visceral and subcutaneous adipose tissue area based on whole-body CT scan and calculated the ratio of VAT to SAT (VSr). Thereafter, the patient population was evenly divided into three groups; respectively VSr value less than 0.4 for the first group (low ratio), 0.4–0.84 for the second group (intermediate ratio), and greater than 0.84 for the third group (high ratio). Considering the other influencing factors; the groups were further divided into subgroups in the respective analysis according to gender (male/female), BMI (<25 or ≥25), and ISS (<26 or ≥26). Result VSr was an independent factor from body mass index (BMI) (r^2^ = 0.003; *p* = 0.553). VSr in male patients was significantly higher (*p* < 0.001). Patients with low VSr had higher ISS scores (*p* = 0.028). Polytrauma patients with higher VSr tended to have lower SIRS scores and significant differences of SIRS score were found on multiple days during the whole hospitalization period. In the low VAT/SAT group, male patients, and patients with BMI greater than 25, both exhibited higher SIRS scores during hospital stay (day 16: *p* = 0.01; day 22: *p* = 0.048 and *p* = 0.011; respectively). During hospitalization, patients with higher ISS score (≥26) in the low VSr group was found to have higher SIRS score (day 16; *p* = 0.007). Over the hospital stay; serum markers of CRP; CK; and leukocyte in patients with low VSr were higher than those in patients in the intermediate and high VSr groups; with significant difference discovered on multiple days (day 16: 0.014; day 22: *p* = 0.048). Conclusion Lower VSr is associated with increased inflammatory response and worse clinical outcome in patients with polytrauma. Furthermore; VSr is an independent factor providing additional information to BMI.

## 1. Introduction

Obesity has been identified as a vital risk factor for cardiovascular diseases [1] and various diseases involving an acute or a long-term chronic inflammatory response, such as asthma [2], Alzheimer’s disease [3], as well as in trauma [4,5] and postoperative patients [6]. Despite an increasing number of studies reporting the positive relationship between obesity and multiple diseases, the link remains controversial. Recent studies reported that overweight and obesity was related to lower risk of mortality for patients in an intensive care unit (ICU) [7,8] and improved functional status after discharge [9]. Another study noted that obese polytrauma patients tend to have milder inflammatory implications than their normal-weight counterparts [10]. The contradictory results suggest an “obesity paradox”, the mechanism of which remains unclear, and the puzzling uncertainty is intensified by the inherent limitations of clinical studies, such as reverse causality or sample group selection bias [11].

Although obesity has been extensively studied by researchers using the body mass index (BMI) as the prevailing standard, the BMI is a raw reflection of the body total adiposity. It cannot distinguish body lean mass from fat mass or consider differently distributed regional adipose tissue, as in visceral adipose tissue (VAT) and subcutaneous adipose tissue (SAT) [12]. Furthermore, VAT is a vital source of proinflammatory mediators, such as tumor necrosis factor (TNF)-α, C-reactive protein (CRP) and interleukin (IL)-6 [13], and anti-inflammatory mediator IL-10 [14]. In contrast, SAT is not involved in the systemic inflammatory response to the same extent. Thus, we postulate that the conflicting results of the previous studies may be attributed to utilizing BMI as the sole index.

After trauma, systemic inflammatory response syndrome (SIRS) is an inflammatory response to blood loss and tissue damage, which may even begin as soon as a half an hour after an injury [15]. In previous studies, the effect of obesity on SIRS in polytrauma patients has been a research topic. However, they yielded contradictory findings [10,16]. To the best of our knowledge, there are few data about SIRS in polytrauma patients analyzed with body composition, especially adipose distribution patterns.

In our study, we utilize the ratio of VAT and SAT as an index [VSr] and investigate the development of SIRS in patients with different values of VSr after polytrauma. Therefore, this study aimed to analyze the development and extend of SIRS of the patients with various body adipose tissue distribution patterns following polytrauma. We hypothesize that patients with higher VSr would show a worse inflammatory response after polytrauma.

## 2. Methods

### 2.1. Patient Population

The retrospective study was conducted at a German Level-I University Trauma Center. We analyzed our medical record database from November 2018 to March 2021. The inclusion criteria were as follows: admission via emergency room (ER), injury severity score (ISS) > 16 points, age ≥ 18 upon admission, availability of the archive to ensure the data extraction from well-documented ICU records and whole-body CT-scan with both sagittal and cross-sectional view of the abdomen and lumbar spine. All the data were collected retrospectively from the electronic medical records archive. Institutional Review Board (IRB) approval was obtained before data collection.

### 2.2. Image Post-Processing—Measurement of the Adipose Tissue Distribution

Following an ATLS^®^ based ER algorithm, all patients with a sufficient cardiopulmonary status were examined with a single-source 64-row CT scanner (Evolution CT, General Electric, Milwaukee, WI, USA). After intravenous bolus injection of iodinated contrast medium, an axial helical whole body polytrauma scan was acquired. Adequate opacification of the vessels was ensured by bolus tracking with SmartPrep (General Electric, Milwaukee, WI, USA).

### 2.3. Body Composition

**Figure 1:**
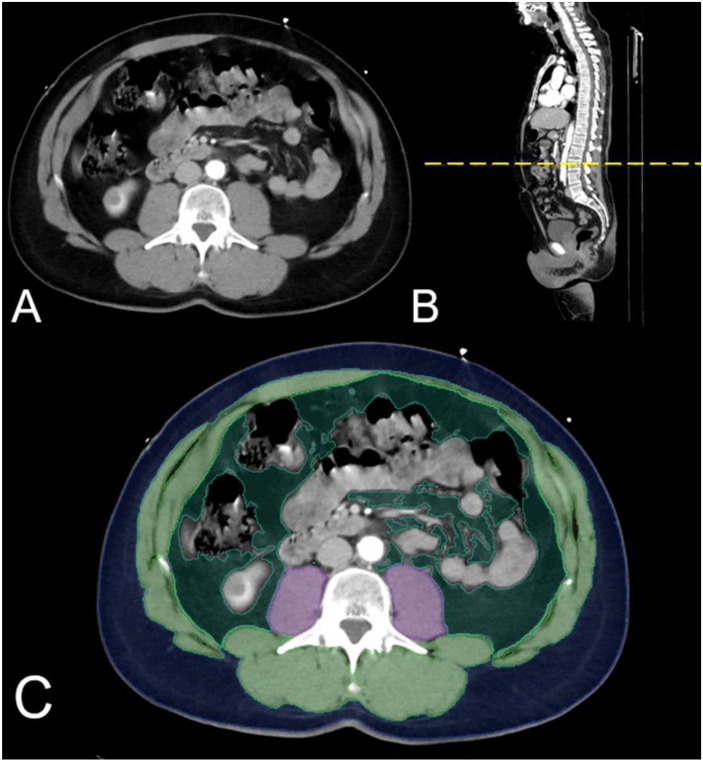
AI based CT Image Segmentation. (**A**,**B**): Example Original axial & sagittal CT images at the level of the third lumbar vertebra. (**C**): CT image with automated tissue segmentation.

For analysis of the body composition parameters, an AI-based automated software tool based on a convolutional neural network, U-net, developed for image segmentation, was used as reported elsewhere (Visage version 7.1., Visage Imaging GmbH, Berlin, Germany) [17]. The network consists of nine blocks: four downsampling blocks, four upsampling blocks, and one in between. The training data consisted of 200 axial CT images at the level of the third lumbar vertebra, and augmentation was applied during training to improve network generalization, also as reported elsewhere [17]. The single tissue compartments were separated into the psoas muscle, skeletal muscle, visceral fat, and subcutaneous fat, each coded in different colours. Other tissues, such as the parenchymal organs (kidney, liver, spleen, intestine, and pancreas), were not segmented. Tissue segmentation was reviewed for correctness and manually corrected if necessary. The area (square centimeters [cm^2^]) and density (Hounsfield unit [HU]) were calculated by the software automatically. The following parameters were derived from the so-called “L3 body composition analysis”: mean density (in HU) of skeletal muscle including the psoas muscle (SMD), and areas (in cm^2^) of skeletal muscle, visceral adipose tissue (VAT), and subcutaneous adipose tissue (SAT) as shown in Figure 1. The scaling of the CT scan window was fixed hence the pixel count was normalized throughout the cohort. The patient cohort was grouped according to the VSr as summarized in Table 1.

### 2.4. Evaluation of Inflammatory Response

The SIRS score was measured daily during the hospitalization or death up to a maximum of 31 days. The score was calculated based on the worst leukocyte count, heart rate, respiratory rate, and body temperature during each hospitalization day (details see Table 2) [18]. In addition, several standard serum markers related to alteration of the inflammation response in an obese population were also collected and analyzed, namely leukocyte count [19], C-reactive protein (CRP) [20], procalcitonin (PCT) [21] and creatine kinase (CK) [22]. Subsequently, mean SIRS scores, max SIRS scores and days of max SIRS score during hospitalization were calculated and analyzed. To evaluate the development of the SIRS, we analyzed the SIRS score measurement in days among the three VSr groups. Based on previous literatures experiences, several potential confounding factors like BMI, gender and ISS were identified, and a correlation analysis was conducted to verify those factors (Supplementary Table S1). Subsequently a stratification was also performed to present the influence of those potential confounding factors. Subsequently, we divided the cohort into subgroups according to the BMI level (≥25, <25), gender (male/female), and ISS (≤26 and >26).

### 2.5. Evaluation of Physiological Parameters

The physiological evaluation was performed during resuscitation on-site and/or upon admission at the ER. Parameters analyzed were: vital signs, complete blood count, coagulation function test and blood gases test (details see Table 1). The level of consciousness was evaluated using the Glasgow Coma Scale (GCS) [23] (Table 1). In addition, the ISS based on AIS (Abbreviated Injury Scale) 2005 was calculated [24,25]. Furthermore, a patient was considered to be in shock if one of the following standards was met: systolic blood pressure (SBP) < 90 mmHg, heart rate to SBP ratio > 1 or base excess < −6 mmol/L [16]. Finally, length of hospitalization, ICU stays, and days of ventilation were recorded.

### 2.6. Statistical Analysis

Data were tested for normal distribution using Kolmogorov–Smirnov test. In the case of non-normal distribution, non-parametric test was conducted. A Kruskal–Wallis test was performed for continuous variables, as was a chi-square test for categorical variables. Moreover, the one-way ANOVA test was used for normally distributed data. Data are reported as mean, standard deviation, median and interquartile range (IQR), depending on the distribution.

Statistical analyses were performed using SPSS version 22.0 (SPSS, Chicago, IL, USA), whereas data acquisition and preprocessing were performed using Python 2.7. A *p* value < 0.05 was considered statistically significant.

## 3. Results

### 3.1. Patient Cohort

Our database search yielded a total of 1200 patients. After applying the inclusion criteria, 𝑛 = 1068 had to be excluded (𝑛 = 796: ISS < 16 or age < 18, 𝑛 = 262: insufficient data available, 𝑛 = 10: no CT-scan). 132 patients met the inclusion criteria and were enrolled in the study. The patient cohort was divided into three groups as mentioned above, resulting in a low (𝑛 = 44), intermediate (𝑛 = 44), and high (𝑛 = 44) ratio group. The overall VSr median was 0.61 (IQR: 0.36, 1.04) (Table 1).

### 3.2. Baseline Characteristics

Demographic and baseline information in each VSr group were comparable, except gender and age. 96 were male, 36 were female, with significantly more male patients in all VSr groups (Table 1). Patients in the intermediate and high VSr group were significantly older than in the low VSr group (Table 1).

### 3.3. Association between Ratio of Visceral Adipose Tissue to Subcutaneous Adipose Tissue (VSr) and Body Mass Index (BMI)

Our analysis revealed moderate positive correlation between VAT and BMI (Pearson r = 0.558, *p* < 0.001) as well as between SAT and BMI (r = 0.602, *p* < 0.001), whereas VSr was, however, not correlated with BMI (r^2^ = 0.003, *p* = 0.553) (Supplementary Materials Table S2). The result suggested that VSr was an independent factor that could provide additional information compared to BMI.

### 3.4. Injury Pattern and Physiological Situation upon Admission

**Figure 2:**
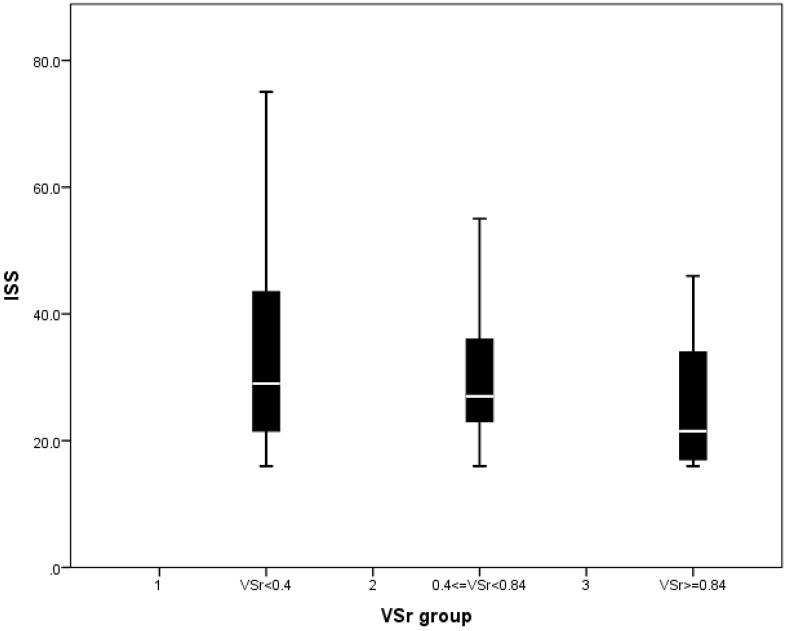
ISS score in VSr groups. Significant difference of ISS score was found between low and high VSr groups (Kruskal Wallis Test, low ratio group (VSr < 0.4) vs. high ratio group (VSr ≥ 0.84), *p* = 0.045).

Analysis of the AIS revealed significant differences among the VSr groups regarding AIS extremities scores (Table 1) and total ISS score (Figure 2). Base excess showed significantly lower values in higher VSr groups (Table 1). ISS did not differ between the (Table 1). Furthermore, we found no significant differences regarding vital signs, complete blood count, coagulation function test, or blood gases when compared between the VSr groups.

### 3.5. Analysis of Systemic Inflammatory Response Syndrome (SIRS) Scores and Clinical Outcomes

**Figure 3:**
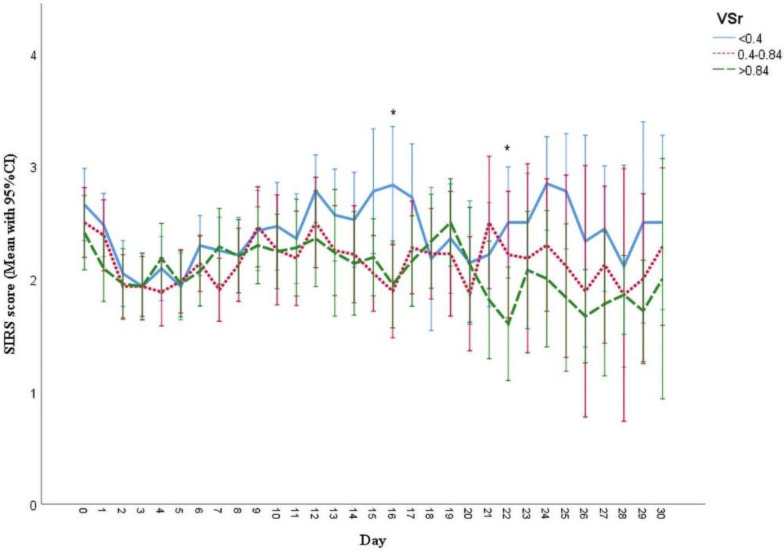
SIRS score during hospital stay in three VSr groups. The SIRS scores of low ratio group tended to be higher than the rest of the two groups. * The differences were found statistically significant on day 16 (low VSr group vs. intermediate VSr, *p* = 0.014; low vs. high, *p* = 0.017), day 22 (low vs. high, *p* = 0.048). Results are shown in mean with 95% CI.

Patients with lower VSr tended to have a higher SIRS score and more ventilation days without significant differences between the groups. In addition, the overall hospitalization and duration of ICU among the three groups were comparable without significant differences. Data are presented in detail in Table 3. Over time, the analysis of the SIRS scores revealed higher values in the low VSr group than the intermediate and high VSr group. However, significant differences could only be detected at day 16 (low vs. intermediate: *p* = 0.014; low vs. high: *p* = 0.017) and 22 (low vs. high: *p* = 0.048), respectively (Figure 3). After adjustment for potential confounding factors as presented in the Supplementary Table S1, the results showed that low VSr has higher SIRS scores than the high VSr (β adjusted = 0.19 [95% CI = 0.07 to 0.30]) (Supplementary Materials Table S1).

### 3.6. Measurement of Creatine Kinase (CK), C-Reactive Protein (CRP) and Leukocyte Count

**Figure 4:**
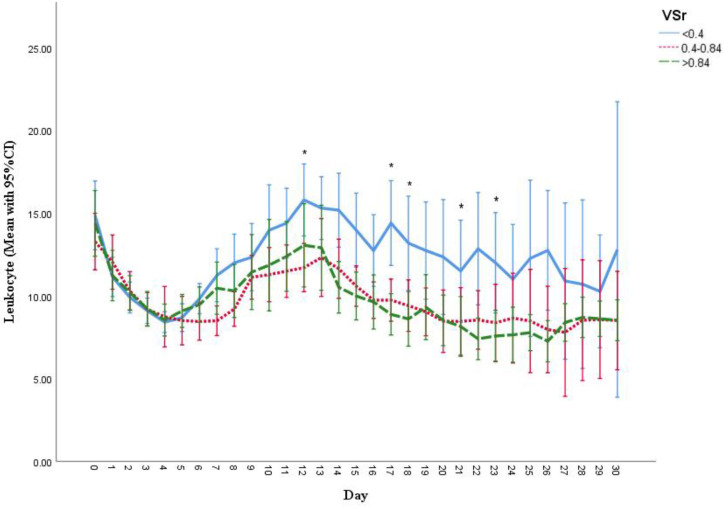
Serum leukocyte level during hospital stay in three VSr groups. * Significant higher level of serum leukocyte level difference was unveiled on day 12 (between low and intermediate VSr group, *p* = 0.012), day 17 (low vs. intermediate, *p* = 0.013; low vs. high *p* = 0.003), day 18 (low vs. high, *p* = 0.029), day 22 (low vs. high, *p* = 0.007) and day 23 (low vs. high, *p* = 0.049). Results are shown in mean with 95% CI.

**Figure 5:**
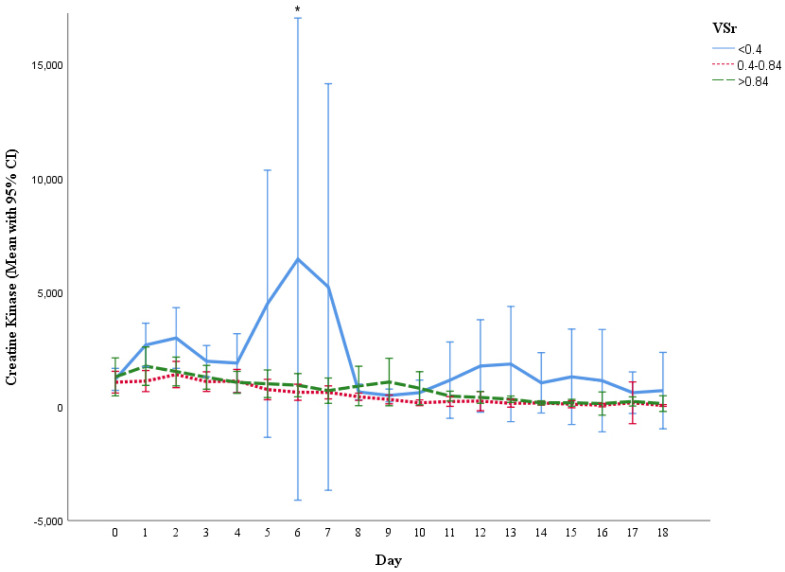
Serum creatine kinase level during hospital stay in three VSr groups. Serum creatine kinase level in low ratio group tends to be higher than the rest of the two groups in all the days of hospital stay. * Significant difference was found on day 6 (between low and high VSr group, *p* = 0.002). Results are shown in mean with 95% CI.

**Figure 6:**
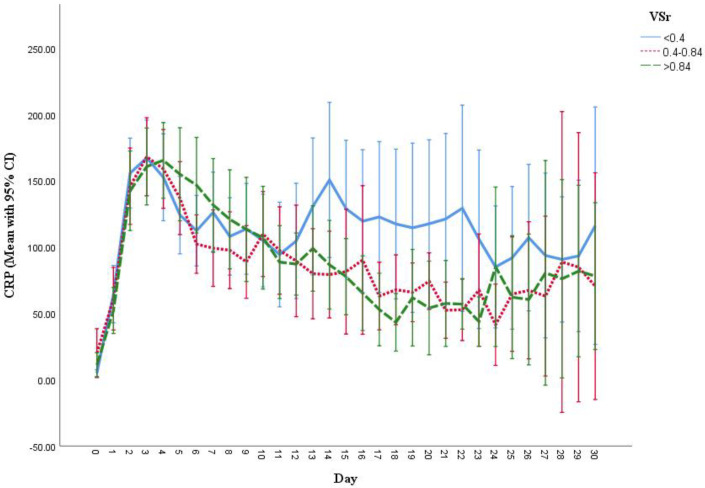
Serum CRP level during hospital stay in three VSr groups. Low VSr group has the trend of higher serum CRP level than the rest of the two groups from the third week of hospital stay. However, no statistically significant difference was found. Results are shown in mean with 95% CI.

Inflammation markers analysis unveiled significant higher leukocytes count in the lower VSr group at day 12 (low vs. intermediate VSr group, *p* = 0.012), day 17 (low vs. intermediate: *p* = 0.003; low vs. high: *p* = 0.003), day 18 (low vs. high: *p* = 0.029), day 22 (low vs. high: *p* = 0.007), and day 23 (low vs. high: *p* = 0.049) (Figure 4). Furthermore, patients in the low VSr group were found to have a significantly higher CK level than the other groups at day 6 (low vs. high: *p* = 0.002.) (Figure 5). Although CRP levels showed no significant differences over time, we observed a trend towards higher CRP levels in the low VSr group starting from the third week after injury (Figure 6).

### 3.7. Analysis of SIRS Scores Stratified by Injury Severity Score (ISS), BMI, and Gender

**Figure 7:**
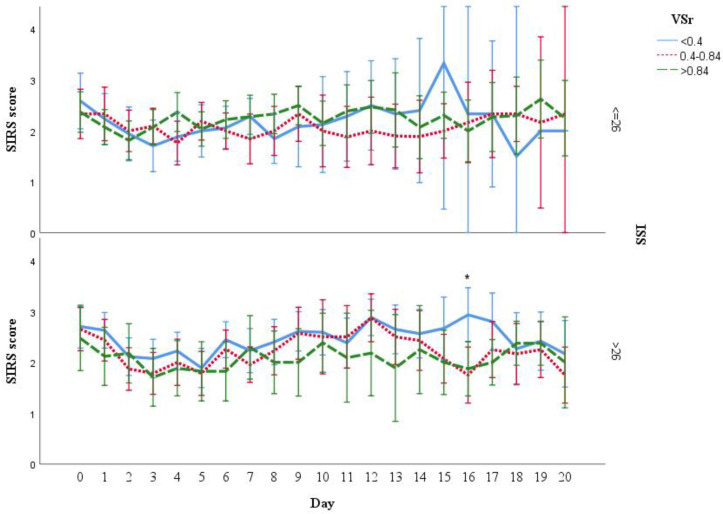
SIRS score during hospitalization grouped by VSr and ISS. In the high ISS subgroup (ISS > 26), SIRS score of patients in lower VSr groups tended to be higher. * Significant higher SIRS score on day 16 (low vs. high VSr group, *p* = 0.007) in high ISS subgroup (ISS ≥ 26). No significant difference of SIRS score was found in low ISS subgroup (ISS ≤ 26). The results are shown as mean with 95% CI.

As ISS scores of the three VSr groups were found to differ significantly (Figure 2), we divided the cohort based on the ISS score (high ISS: >26 and low ISS ≤ 26) and conducted our analysis separately on both groups to address this confounder. Analysis showed that in the high ISS group, patients with low VSr had the tendency towards higher SIRS score over time. However, significant differences were only found at day 16 (low vs. high: *p* = 0.007, Figure 7).

**Figure 8:**
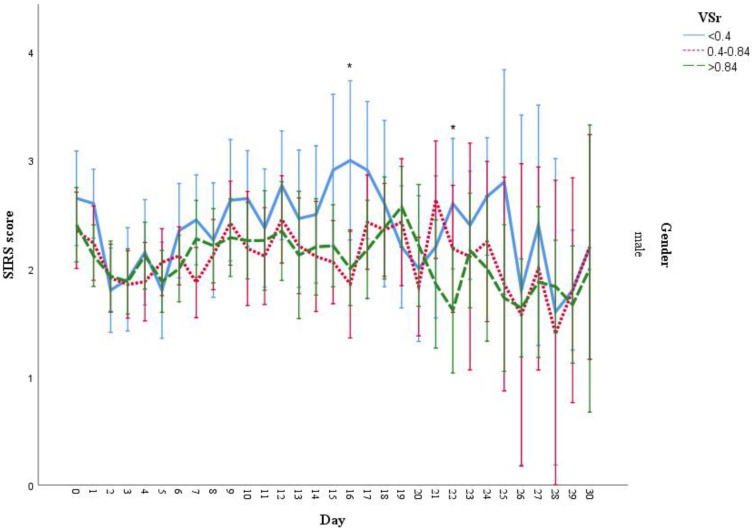
SIRS score during hospitalization grouped by VSr and gender. * Significant difference of SIRS score was found on the day 16 (*p* = 0.01) and day 22 (*p* = 0.048) in males between low and high VSr group. Result are presented as mean with 95% CI. Due to the limited availability of data and subsequently inadequate statistical power for female patients under three VSr groups, the chart for female was not presented.

Female patients had significant lower VAT (*p* < 0.001), VSr (*p* < 0.001), and muscle area (*p* < 0.001) (Table 4). Regarding gender differences, we found that male patients with low VSr ratio had a higher SIRS score, with significant differences found at day 16 (*p* = 0.01) and day 22 (*p* = 0.048) (Figure 8). Due to low patient numbers, we did not perform this analysis in the female cohort. 

**Figure 9:**
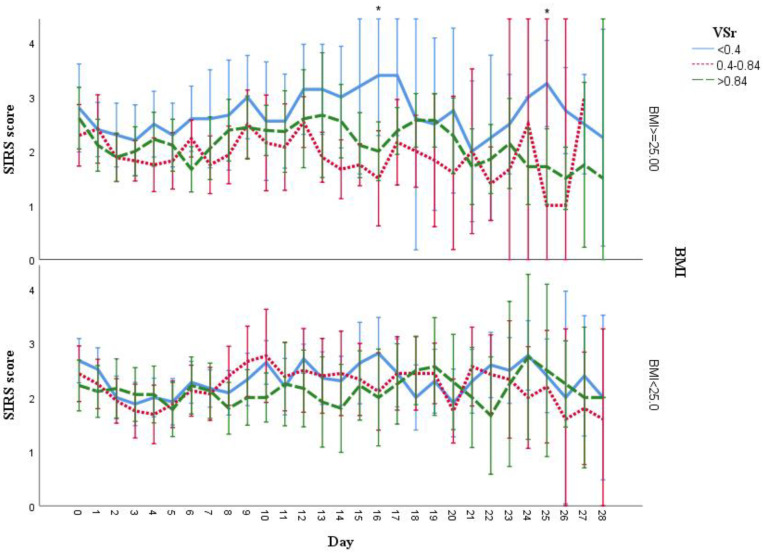
SIRS score during hospitalization grouped by VSr and BMI. In the subgroup BMI ≥ 25, patients with lower VSr had a higher trend of SIRS score during hospitalization. * Significant difference was found at day 16 (low vs. intermediate VSr group, *p* = 0.01) and day 25 (low vs. intermediate, *p* = 0.011; intermediate vs. high, *p* = 0.044). No significant difference was found among VSr groups in patients with BMI < 25. Results are shown as mean with 95% CI.

Analysis of the subgroups stratified based on BMI revealed an increasing trend of SIRS score of patients in the low VSr group with significant differences found at day 16 (*p* = 0.01) and day 25 (low vs. intermediate VSr group: *p* = 0.011, intermediate vs. high VSr group: *p* = 0.044) (Figure 9).

## 4. Discussion

Studies have pointed out a strong link between obesity and worse clinical outcomes for trauma patients in recent years. For example, obese trauma patients showed increased mortality, morbidity and higher complication rates [26,27,28]. Furthermore, an adverse pro-inflammatory state was also seen in obese patients compared to regular weight counterparts suffering trauma; in this study, the researchers found a positive relationship between higher BMI and lower max SIRS during hospitalization [10]. Similarly, when compared with non-obese polytrauma patients, patients with BMI > 30 kg/m^2^ had significantly higher IL-6, CRP and were found to be more susceptible to multiple organ dysfunction syndrome (MODS) [29]. However, BMI as a surrogate parameter could not unravel whether the consequences were due to differently distributed fat tissue because BMI does not provide information about body composition. This was yet noted by several scholars and considered as one vital cause of the “obesity paradox” [30,31]. 

Our study evaluated the potential influence of the distribution of fat tissue on the development of inflammatory response in polytrauma patients with the help of CT-based artificial intelligent measurement of visceral and subcutaneous fat tissue. Therefore, the SIRS score and standard serum inflammation parameters were analyzed. We found a trend that patients with higher VSr had a minor SIRS score compared to lower VSr. However, this trend was not observed in several subgroup analyses (female, patients with BMI < 25 and ISS < 26). Thus, these findings did not support our primary hypothesis. Additionally, we found that leukocyte count, CK and CRP levels followed a similar pattern: patients with higher VSr had minor standard inflammation markers serum levels. 

Regarding inflammatory response following trauma in obese patients, Pisitsak et al., found higher pro-inflammatory cytokine IL-8 and lower anti-inflammatory maker IL-10 levels in trauma patients with higher VSr [32]. Collier et al., however, reported that VSr is not associated with increased inflammatory profiles and clinical outcomes [33]. Independent of trauma cases, visceral obesity was associated with higher CRP levels following surgery for oesophagus adenocarcinoma [34]. Additionally, previously published studies reported that higher VSr was associated with an increased inflammatory response in colorectal resection surgery [35] and esophagectomy [36]. These results are contradictory. One possible explanation might be the limited population sample to analyze inflammation markers in all studies available.

Furthermore, fat tissue reacts in the early post-traumatic immune and inflammation responses [29]. Therefore, obese patients might be in a pro-inflammatory state, indicated by an elevated baseline of blood levels of inflammatory markers [37]. However, when combined with the limitation of the patient cohort size, this circumstance would diminish the possibility of blood sample comparison because of insufficient power of the subgroups. 

Moreover, due to the advancements in the ICU resuscitation protocol during the past decades, the measurement of SIRS score and inflammation markers might be altered and, therefore, biased. However, during our study period, the protocol in our ICU remained unchanged. 

Nonetheless, the issues mentioned above are not suitable for clarifying why we found higher SIRS scores in patients with lower VSr, because the mechanism remained unclear. However, one possible explanation of our findings could be the so-called browning of fat tissue: basically, fat tissue can be subdivided into brown adipose tissue (BAT) and white adipose tissue (WAT). These two interact with each other to maintain a balance between energy storage and consumption [38]. Moreover, fat tissue not only stores energy but also links to metabolism and immunity. In fat tissue, numerous cell types are found, secreting more than 100 different cytokines, adipokines and chemokines [39] that play a vital role in the inflammation and immunological response. Between the two types of fatty tissue, BAT was found to be less prone to developing inflammation than WAT [38], and BAT can alleviate fat tissue inflammation by attenuating pro-inflammation cytokines [40]. Previously published data demonstrate that VAT has a higher expression of browning genes than SAT [41].

Furthermore, it was reported that surgery and trauma could trigger the browning of fat tissue [42]. Additionally, cold stimulation [43] and burning injury [44] can also activate the browning of fat tissue. However, the certification or falsification of such a link still requires further studies on the underlying molecular and cellular mechanisms.

There are several limitations to our study. First, it is retrospective, with all its known restrictions. Second, the SIRS and ISS scores were subjective from independent researchers, which were prone to be biased. Third, the cutoff point of VSr based on radiological imaging has not been validated yet. More data are required to reach a consensus of a VSr cutoff value to define visceral adiposity. Fourth, a selection bias exists due to limited available data. In addition, although statistical significance was found in various analyses, the patient cohort was still relatively small, which may reduce the value of the conclusions drawn. 

## 5. Conclusions

Lower VSr may be a sign of worse inflammation response in patients suffering a polytrauma. Further investigations are needed to confirm our findings. In addition, potential mechanisms should also be studied to establish clinical countermeasures to alleviate the detriment.

## Figures and Tables

**Table 1:** Baseline Characteristics of the patient population upon admission.

Characteristics	Total	VSr < 0.4	VSr 0.4–0.84	VSr > 0.84	*p*
Number of patients	132	44	44	44	
Gender (male/female)—*n* (%)	96/36 (72.7%, 27.3%)	20/24 (45.5%, 54.5%)	34/10 (77.3%, 22.7%)	42/2 (95.5%, 4.5%)	<0.001
Age (years) *	55.4 (20.7)	40.5 (21.3)	59.5 (20.9)	53.8 (12.2)	<0.001
Survived—*n* (%)	122 (92.4%)	42 (31.8%)	41 (31.1%)	39 (29.5%)	0.469
VSr **	0.61 (0.36, 1.04)	0.24 (0.13, 0.37)	0.61 (0.51, 0.72)	1.27 (1.03, 1.77)	<0.001
AIS head	3.0 (2.0, 4.0)	3.0 (1.0, 4.0)	3.0 (2.0, 4.0)	4.0 (3.0, 4.0)	0.170
AIS face	0 (0, 1.0)	0 (0, 1.5)	0 (0, 2.0)	0 (0, 0)	0.099
AIS thorax	3.0 (0, 4.0)	3.0 (0, 4.0)	3.0 (0, 4.0)	2.0 (0, 3.0)	0.153
AIS abdomen	0 (0, 2.0)	0 (0, 3.0)	0.5 (0, 2.5)	0 (0, 2.0)	0.396
AIS extremities	2.0 (0, 3.0)	2.0 (2.0, 4.0)	2.0 (0, 3.0)	0 (0, 2.0)	<0.001
AIS external	0 (0, 0)	0 (0, 0.5)	0 (0, 0)	0 (0, 0)	0.380
ISS	27.0 (20.0, 36.0)	29.0 (21.5, 43.5)	28.0 (23.0, 37.0)	21.5 (17.0, 34.0)	0.045
Glasgow coma scale (GCS)	3.0 (3.0, 9.0)	3.0 (3.0, 10.0)	3.0 (3.0, 5.0)	3.0 (3.0, 10.0)	0.764
Shock (yes/no)	97/35	30/14	32/12	35/9	0.478
Systolic pressure (mmHg)	90.4 (17.1)	90.3 (17.8)	91.9 (17.6)	88.9 (16.3)	0.720
Diastolic pressure (mmHg)	50.1 (11.2)	46.8 (11.9)	51.7 (10.1)	51.8 (11.1)	0.058
Heart rate (/min)	105.1 (21.7)	103.6 (18.6)	105.7 (22.4)	105.9 (24.2)	0.856
Haemoglobin (g/dL)	11.2 (2.3)	10.8 (2.2)	11.3 (2.2)	11.6 (2.5)	0.313
Platelet count (/nL)	201.2 (83.3)	211.2 (90.3)	200.9 (89.0)	191.4 (69.6)	0.539
Prothrombin time (%)	63.3 (25.4)	62.1 (25.2)	62.2 (25.1)	66.2 (26.9)	0.820
PH	7.31 (0.11)	7.32 (0.12)	7.30 (0.10)	7.31 (0.11)	0.627
Base excess	−4.4 (4.0)	−3.1 (3.9)	−4.7 (3.6)	−5.5 (4.1)	0.013
INR	1.17 (1.08, 1.36)	1.19 (1.08, 1.35)	1.16 (1.06, 1.42)	1.17 (1.10, 1.34)	0.958
APTT	35.4 (30.8, 45.2)	36.25 (30.80, 45.40)	35.30 (30.90, 49.80)	33.70 (30.60, 42.20)	0.815

AIS: abbreviated injury scale; ISS: injury severity score; INR: international normalized ratio; APTT: activated partial thromboplastin time. * Data are given as the mean (SD) and ANOVA was performed. ** Data are given as the median (IQR) and Kruskal-Wallis test was performed.

**Table 2:** SIRS score calculation.

Criteria *	Primary Criteria	Alternative Criteria
Leukocyte count	>12,000 or <4000/mL	10% immature forms or bands
Heart rate	>90/min	N/A
Respiratory rate	>20/min	partial pressure of CO_2_ < 32mmHg
Body temperature	>38 °C or <36 °C	N/A

* Each criteria met in the primary or alternative criteria column is counted as 1 point into the total SIRS score.

**Table 3:** Analysis of the SIRS scores and clinical outcomes for the 3 VSr groups.

	Total	VSr < 0.4	VSr 0.4–0.84	VSr > 0.84	*p*
Mean SIRS score—Mean (SD)	2.16 (0.57)	2.26 (0.59)	2.11 (0.57)	2.11 (0.56)	0.354
Max SIRS score—Mean (SD)	3.48 (0.71)	3.57 (0.73)	3.39 (0.75)	3.48 (0.66)	0.494
Days of max SIRS score(d)—Median (IQR) *	3.0 (1.0, 4.0)	2.0 (1.0, 4.0)	3.0 (2.0, 4.0)	3.0 (1.5, 4.0)	0.387
Hospitalization(d)—Median (IQR) *	20.0 (14.0, 28.0)	18.5 (12.0, 27.0)	22.0 (16.0, 28.0)	19.0 (13.0, 28.0)	0.335
ICU stay(d)—Median (IQR) *	14.0 (10.0, 25.0)	14.0 (10.0, 25.0)	15.5 (11.5, 24.5)	14.0 (10.0, 26.5)	0.686
Ventilator duration(d)—Median (IQR) *	11.0 (5.0, 22.0)	7.5 (4.0, 21.5)	11.0 (7.0, 21.0)	12.0 (5.0, 23.0)	0.352

* One-way ANOVA test, data are given as the mean (standard deviation). Kruskal-Wallis test, data are given as the median (IQR).

**Table 4:** Adipose tissue distribution in two genders.

	Female	Male	*p*
VAT (pixel count)	4287.5 (2121.5, 13,278.0)	14,460.0 (7282.0, 26,344.5)	<0.001
SAT (pixel count)	16,087.5 (9099.5, 31,848.5)	18,618.0 (10,488.0, 24,650.0)	0.85
VSr	0.29 (0.14, 0.51)	0.74 (0.46, 1.19)	<0.001
Muscle (pixel count)	13,003.0 (11,837.0, 15,573.5)	17,322.0 (15,584.5, 21,016.5)	<0.001

Median (IQR). Strong difference of VAT, muscle and VSr was found between gender.

## Data Availability

The data presented in this study are available on request from the corresponding author. The data are not publicly available due to privacy.

## Supplementary Materials

The following are available online at https://www.mdpi.com/article/10.3390/life11111243/s1, Table S1: Estimated correlations between ratio of visceral adipose tissue to subcutaneous adipose tissue (VSr) and the systemic inflammatory response syndrome (SIRS) scores after adjustment confounding factors. Table S2: Linear regression analysis of visceral adipose tissue (VAT), subcutaneous adipose tissue (SAT), VSr and BMI.
