# Supplementary material for: The Effect of Fat Distribution on the Inflammatory Response of Multiple Trauma Patients—A Retrospective Study
Source: Life (Basel). 2021 Nov 16;11(11):1243. doi: 10.3390/life11111243 (PMC8625240; doi:10.3390/life11111243)
Supplement: Supplementary file 1 [file life-11-01243-s001.zip › life-1441110-supplementary.pdf]

life

MDPI

Article

The Effect of Fat Distribution on the Inflammatory Response of Multiple Trauma Patients—A Retrospective Study

Zhaoxiong Chen [1], Silvan Wittenberg [1], Timo Alexander Auer [2], Maxim Bashkuev [3], Pimrapat Gebert [4], Uli Fehrenbach [5], Dominik Geisel [5], Frank Graef [1], Sven Maerdian [1*] and Serafeim Tsitsilonis [1*]

1. Center for Musculoskeletal Surgery, Corporate Member of Freie Universität Berlin, Humboldt-Universität zu Berlin, Berlin Institute of Health, Charité - Universitätsmedizin Berlin, 10115 Berlin, Germany; zhaoxiong.chen@charite.de (Z.C.); silvan.wittenberg@charite.de (S.W.); frank.graef@charite.de (F.G.)
2. Clinic for Radiology, Corporate Member of Freie Universität Berlin, Humboldt-Universität zu Berlin, Berlin Institute of Health, Charité - Universitätsmedizin Berlin, 10115 Berlin, Germany; timo-alexander.auer@charite.de
3. Julius Wolff Institute for Biomechanics and Musculoskeletal Regeneration, Charité - Universitätsmedizin Berlin 10115 Berlin, Germany; maxim.bashkuev@charite.de
4. Institute of Biometry and Clinical Epidemiology, Charité - Universitätsmedizin Berlin, 10115 Berlin, Germany; pimrapat.gebert@charite.de
5. Clinic for Radiology, Corporate Member of Freie Universität Berlin, Humboldt-Universität zu Berlin, Charité - Universitätsmedizin Berlin, 10115 Berlin, Germany; uli.fehrenbach@charite.de (U.F.); dominik.geisel@charite.de (D.G.);

* Correspondence: sven.maerdian@charite.de (S.M.); serafeim.tsitsilonis@charite.de (S.T.)

Citation: Chen, Z.; Wittenberg, S.; Auer, T.A.; Bashkuev, M.; Gebert, P.; Fehrenbach, U.; Geisel, D.; Graef, F.; Maerdian, S.; Tsitsilonis, S. The Effect of Fat Distribution on the Inflammatory Response of Multiple Trauma Patients—A Retrospective Study. *Life* **2021**, *11*, 1243. https://doi.org/10.3390/life11111243

Academic Editors: Claudia Neunaber, Milena Fini and Paolo Cinelli

Received: 14 October 2021 Accepted: 12 November 2021 Published: 16 November 2021

Publisher's Note: MDPI stays neu-tral with regard to jurisdictional claims in published maps and institu-tional affiliations.

Copyright: © 2021 by the authors. Licensee MDPI, Basel, Switzerland. This article is an open access article distributed under the terms and conditions of the Creative Commons Attribution (CC BY) license (http://creativecommons.org/licenses/by/4.0/).

Table S1. Estimated correlations between VSr and the SIRS scores after adjustment confounding factors.

	Unstandardized Coefficient (95%CI)		*p*-value
	β_{crude} (95%CI)	$\beta_{adjusted}$ (95%CI)	
**Time (Day)**	0.003 (-0.002, 0.008)	0.001 (-0.004, 0.006)	0.747
Male	-0.13 (-0.23, -0.04)	-0.05 (-0.17, 0.06)	0.350
VSr			
<0.4	0.25 (0.15, 0.35)	0.19 (0.07, 0.30)	0.002
0.4-0.8	0.04 (-0.06, 0.13)	-0.09 (-0.20, 0.02)	0.108
>0.8	Reference	Reference	
BMI	-0.008 (-0.02, 0.002)	0.003 (-0.008, 0.014)	0.575
ISS	0.01 (0.007, 0.013)	0.007 (0.003, 0.010)	<0.001

Table S2. Linear regression analysis of VAT, SAT, VSr and BMI

	VAT	SAT	Vsr
BMI	r^2=0.311, p<0.001	r^2=0.362, p<0.001	r^2=0.003, p=0.553
